# Guillain-Barré Syndrome in Adults in a Decade: The Largest, Single-Center, Cross-Sectional Study From the Kingdom of Saudi Arabia

**DOI:** 10.7759/cureus.40995

**Published:** 2023-06-26

**Authors:** Nada A AlKahtani, Joud A Alkhudair, Nora Z Bensaeed, Yara S Alshammari, Rahaf F Alanazi, Ismail A Khatri, Nazish Masud

**Affiliations:** 1 College of Medicine, King Saud Bin Abdulaziz University for Health Sciences, Riyadh, SAU; 2 Department of Neurology, King Abdullah International Medical Research Center, Riyadh, SAU; 3 Division of Neurology, Department of Medicine, King Abdulaziz Medical City, Ministry of National Guard Health Affairs, Riyadh, SAU; 4 Department of Biostatistics, Epidemiology and Environmental Health Sciences, Georgia Southern University, Statesboro, USA

**Keywords:** acute motor axonal neuropathy (aman), saudi arabia, outcome, autoimmunity, neurophysiology, peripheral neuropathy, adult, flaccid paralysis, guillain-barré syndrome

## Abstract

Background:Guillain-Barré Syndrome (GBS) is the most common cause of acute, usually post-infectious, peripheral neuropathy resulting in a symmetrical, ascending paralysis. We evaluated the clinical and neurophysiological features, treatment, and outcomes of patients with GBS in our center.

Methods: A retrospective chart review on patients with GBS admitted to King Abdulaziz Medical City, Riyadh, Saudi Arabia, from January 2011 to December 2020. Data were analyzed using JMP statistical software version 15 pro.

Results:A total of 86 patients who met the criteria were included, 55 (64%) were males, with a mean age of 49.5+/-17.5 years. Antecedent infection was reported in 53 (61.6%), 51 (62.2%) presented within one week of symptoms onset. Ascending weakness was seen in 55 (70.5%), while 70 (81.4%) had areflexia. Acute motor axonal neuropathy (AMAN) was the commonest electrophysiological type of GBS in 41 (51.9%) patients. Albuminocytologic dissociation was seen in 48 (57%) who had lumbar puncture. Nearly half, 41 (47.7%) were admitted to the intensive care unit (ICU). Seventy (81.3%) were treated with intravenous immunoglobulin. There was no significant difference in the clinical presentation, management, ICU requirement, and discharge disposition between males and females. Females were more likely to have a higher disability at discharge (p=0.01). Patients younger than 60 years were more likely to require ICU admission (p=<0.01).

Conclusion: Our patients with GBS were slightly older than previously reported from the region. AMAN was the commonest type of GBS. Younger patients were more likely to need ICU admission, whereas females were more likely to have a more severe disability.

## Introduction

Guillain-Barré syndrome (GBS) is a rare autoimmune post-infectious disease of the peripheral nervous system that can affect either the myelin sheath that encapsulates the nerve, the nerve fiber itself, or both simultaneously. It typically presents as an acute or subacute form of ascending weakness and numbness, usually following a microbial infection [1]. Being a rare disorder, the estimated incidence of GBS reaches up to two per 100,000 persons per year, and it is found to have slightly higher male predominance [1,2].

The heterogeneity of GBS pathogenesis is thought to be attributed to antibodies cross-reacting with distinct nerve targets that are produced in response to infectious organisms, including Campylobacter jejuni, Cytomegalovirus, Epstein-Barr virus, Mycoplasma pneumoniae, Hemophilus influenzae, and Zika virus [3-5]. Recently, COVID-19 has also been reported to be a trigger of GBS [6]. Cross-reactivity is probably facilitated through molecular mimicry, which is the immunopathogenic mechanism hypothesized to explain the damage to peripheral nerve fibers and surrounding myelin sheath in GBS [7]. The symptoms of the disease usually begin as a tingling sensation in the limbs that later progresses to muscle weakness. Disability peaks in two to four weeks and may result in complete flaccid paralysis over time. The consequences of the resulting neuromuscular dysfunction are characterized by generalized paralysis symmetrically ascending from the lower limbs to the upper limbs, autonomic disturbances provoking cardiac arrhythmia, and blood pressure fluctuations. Although it ordinarily involves the upper and lower limbs, weakness can possibly extend to the cranial nerves, which can affect the diaphragm and pharyngeal muscles, manifesting as respiratory failure and dysphagia [1,2]. GBS has various clinical and neurophysiological subtypes, with the most commonly presenting variants being acute inflammatory demyelinating polyneuropathy (AIDP) and acute motor axonal neuropathy (AMAN). Other variants include acute motor and sensory axonal neuropathy (AMSAN), Miller-Fisher syndrome (MFS), and even more rare, acute pandysautonomia [8]. The diagnosis of GBS mainly relies on a high index of clinical suspicion. Additional confirmatory investigations include lumbar puncture (LP), nerve conduction studies (NCS), and electromyography (EMG), which are crucial to exclude other possible diagnoses. Albuminocytologic dissociation (ACD), an elevation in cerebrospinal fluid (CSF) proteins without pleocytosis, is detectable through LP in up to 71.2% of GBS cases [9]. Neurophysiological abnormalities include prolongation or absence of F-wave, which can be seen in nearly 84% of GBS patients, reduction in motor nerve conduction velocity seen in approximately 52% of GBS cases. A variety of other neurophysiological abnormalities, including conduction block, temporal dispersion, and decreased amplitude, can be seen depending on the type of GBS [6,9,10]. Electrodiagnostic studies may help in differentiating various types of GBS and can help in determining patients' prognosis. Once the diagnosis is established, appropriate treatment regimens are chosen. The standard of care treatment for GBS includes intravenous immunoglobulin (IVIG) or plasma exchange (PE) [11]. Either PE therapy or IVIG can be initiated within four weeks of disease onset. As a consequence of progressive weakness affecting respiratory muscles, patients may need to be monitored in ICU at an early stage. Non-invasive, and sometimes mechanical ventilation may be needed as respiratory failure is seen in 20%-30% of cases. Most patients require physical rehabilitation. Prevention of deep vein thrombosis (DVT) using mechanical or pharmacological prophylaxis is crucial, as disease progression leaves patients in a non-ambulatory state.

There are few single-center studies and a recent multicenter study from Saudi Arabia describing the clinical characteristics of GBS [12-15]. Although GBS patients can share similar demographic, clinical, and treatment patterns, regional variations of the disease's natural history are worth determining, as Saudi Arabia is the largest country in the Middle East [1,2]. The aim of our study was to explore regional demographic trends, clinical patterns, prevalent neurophysiological subtypes, preferred treatment modalities used, and outcomes of GBS among adult patients at King Abdulaziz Medical City (KAMC), Ministry of National Guard Health Affairs (MNGHA), in Riyadh, Kingdom of Saudi Arabia (KSA). This study is promising enough to provide a valuable contribution to the national GBS database to better comprehend vital demographic, clinical, and neurophysiological patterns of the disease.

## Materials and methods

Study design and participants

The study was approved by the institutional review board of King Abdullah International Medical Research Center (KAIMRC), Riyadh with approval number SP20/250/R. This was a retrospective, cross-sectional, chart review; that investigated demographic, clinical as well as neurophysiological characteristics, treatments, and outcomes of adult patients with GBS at tertiary care hospital in Riyadh, KSA from January 2011 to December 2020. The patients were identified from hospital discharge records using ICD codes. Adult patients (≥18 years old), of both genders were included. The investigators conducted a thorough review of the patient’s medical records, both paper charts and electronic medical records. Patients’ eligibility was maintained through implementing simple inclusion criteria; adult patients (≥18 years old), both genders, with an established diagnosis of GBS, were the target of the study. The diagnosis of GBS was confirmed by trained neurologists based on clinical presentation, physical examination, and several diagnostic studies including CSF studies, NCS, and serological markers. In patients with unclear diagnoses, an MRI of the brain and spine was performed to exclude any central causes of patients’ symptoms. All patients with a final diagnosis of GBS during the study period were included to exclude selection bias. Patients with acute paralysis whose final diagnosis was other than GBS (e.g. chronic inflammatory demyelinating polyradiculoneuropathy, transverse myelitis, metabolic neuropathies, etc.) were excluded.

Data collection and main variables

Each patient was assigned a study-specific number for confidentiality. The data was collected by co-investigators who were medical students. The data collection sheet was pre-approved by the IRB. Demographic, clinical, neurophysiological, treatment, and outcome data were obtained. The investigations conducted on the patients, particularly the results of CSF, NCS, and EMG were recorded. The type of GBS was determined based on the results of neurophysiological studies and serological tests. Neurological and non-neurological complications were recorded. Discharge outcomes in terms of severity of disability and discharge disposition were recorded. Hughes GBS Disability Scale and modified Rankin Scale scores were used to determine the severity and disability. Age was categorized into two groups below 60 and above 60 years of age. When outpatient follow up visit data was available, the severity of disability was recorded at three months, six months and one year. 

Statistical analysis

Microsoft Excel software was used for data entry. Data were analyzed using JMP statistical software version 15 pro. Categorical data were presented as frequencies and percentages, and numerical data were presented as means with standard deviation. Pearson's chi-square, Fisher's exact test, and binary logistic regression were employed to analyze the collected data. Binary logistic regression models were constructed to adjust for any confounding effect between age, gender, ICU length of stay, duration of symptoms, length of stay in hospital to identify the variables significantly associated with predictors of poor outcome among adult GBS patients. A p-value of <0.05 was considered significant.

## Results

A total of 86 adult cases were admitted to the KAMC with a diagnosis of GBS. Males constituted 55 (64.0%) of the sample, whereas 31 (36.0%) were females. The mean age at onset of the disease was 49.5 +/-17.5 years. Prior to the onset of GBS symptoms, 53 (61.6%) patients reported a history of infection. The demographic features, comorbid conditions, and antecedent infections are shown in Table 1. The majority of patients had comorbid medical conditions. The prior neuromuscular disease was reported by six (7.0%) patients, whereas seven (8.1%) had a prior history of neuropathy.

**Table 1:** Demographic features, premorbid conditions, and history of antecedent infection in patients with Guillain-Barré syndrome (N=86)

Variables	Categories	N (%)
Gender	Male	55 (64.0)
	Female	31 (36.0)
Age (in years)	Mean±SD	49.5±17.5
Nationality	Saudi	73 (84.9)
	Non-Saudi	13 (15.1)
Marital Status	Married	70 (81.4)
	Single	14 (16.3)
	Divorced	2 (2.3)
Antecedent infection	Respiratory tract infection	28 (32.6)
	Urinary tract infection	8 (9.3)
	Diarrhea and other infections	17 (19.8)
Comorbid Medical Conditions	Hypertension	38 (44.2)
	Diabetes mellitus	26 (30.2)
	Coronary artery disease	11 (12.8)
	Previous neuromuscular disease	6 (7.0)
	Previous neuropathy	7 (8.1)
	Hypothyroidism	7 (8.1)
	Smoking	10 (11.6)
	Recent vaccination	1 (1.2)
	Recent surgery	4 (4.7)

Almost two-thirds, 51 (62.2%) patients presented within one week of symptoms onset. Ascending, symmetric weakness of all four limbs was the commonest presentation. All 4 limbs were involved in 69 (87.3%) patients, followed by involvement of both legs in seven patients (8.9%), two (2.5%) patients had weakness only in one leg, and one (1.3%) had weakness involving only one arm. Forty-eight (60.0%) patients complained of numbness, 31 (63.3%) of them had numbness in all the limbs, nine (18.4%) in both legs, five (10.2%) in both arms, three (6.1%) reported numbness in one leg, and only one (2.0%) reported numbness involving one arm. Back pain and limb pain were common. Facial weakness was seen in 28 (32.6%) patients, whereas ophthalmoparesis/ophthalmoplegia was seen in 15 (17.4%), whereas autonomic dysfunction was seen in 17 (20.2%) patients. Labile blood pressure in 11 (12.9%) and cardiac arrhythmia in 15 (17.7%) cases were the commonest autonomic abnormalities. Most patients were found to have reflexes abnormalities, 70 (81.4%) of patients had areflexia/hyporeflexia, whereas only one (1.2%) patient had hyper-reflexia. Respiratory weakness developed in 24 (27.9%) and dyspnea/difficulty breathing in 30 (34.9%) cases (Table 2).

**Table 2:** Clinical, neurophysiological and serological features of patients with Guillain-Barré syndrome.

Variables	Categories	N (%)
Symptoms duration before presentation	< 1 week	51 (62.2%)
	1-2 weeks	17 (20.7%)
	3-4 weeks	8 (9.8%)
	> 4 weeks	6 (7.3%)
Weakness	Present	76 (88.4%)
Pattern of weakness	Symmetric	64 (83.1%)
	Asymmetric	12 (15.6%)
	Ascending	55 (70.5%)
	Descending	11 (14.1%)
	Undetermined	12 (15.4%)
Numbness		48 (60.0%)
Facial weakness		28 (32.6%)
Ophthalmoparesis/Ophthalmoplegia		15 (17.4%)
Pupillary abnormalities		5 (5.8%)
Diplopia		11 (12.8%)
Dysarthria		18 (20.9%)
Dysphagia		17 (19.8%)
Areflexia/hyporeflexia		70 (81.4%)
Hyperreflexia		1 (1.2%)
Respiratory weakness		24 (27.9%)
Limb ataxia		25 (29.4%)
Gait ataxia		18 (23.1%)
Autonomic dysfunction		17 (20.2%)
Bladder involvement		6 (7.1%)
Bowel involvement		10 (12.1%)
Back pain		32 (38.6%)
Limb pain		25 (29.4%)
Cerebrospinal Fluid		
Cell Count	0 – 5 cells	60 (89.6%)
	6 – 10 cells	4 (6.0%)
	>10 cells	3 (4.5%)
Protein	Normal	23 (33.3%)
	Elevated	46 (66.7%)
Neurophysiological Findings		
Nerve Conduction Studies	Normal	7 (8.9%)
	Abnormal	72 (91.1)
Electromyography	Normal	10 (11.6)
	Abnormal	44 (51.2%)
	Not performed	32 (37.2%)
Serological Testing		
Antibodies to Campylobacter	Normal	6 (7.0%)
	Not done	80 (93%)
GBS specific antibodies	Normal	9 (10.5%)
	Abnormal	7 (8.1%)
	Not done	70 (81.4%)

Management summary

**Figure 1:**
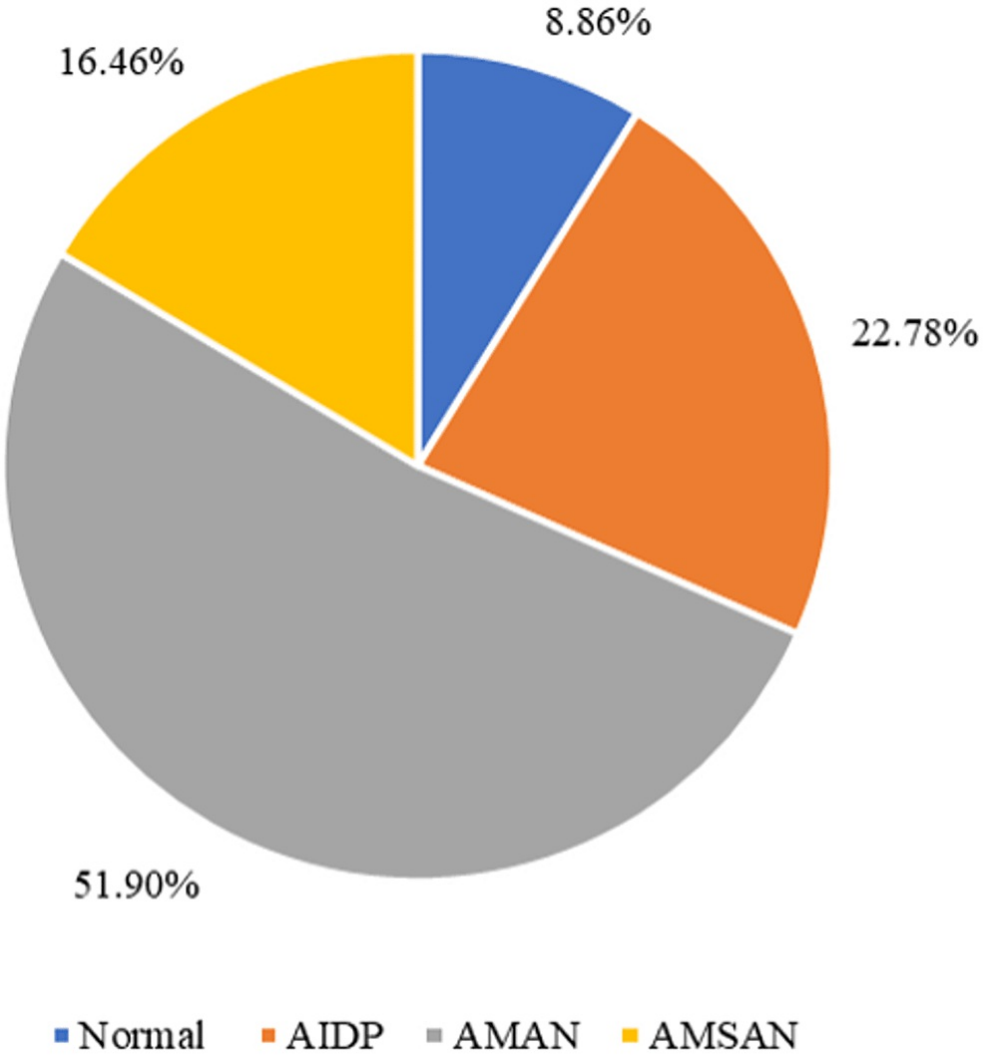
Neurophysiological patterns of Guillain-Barré syndrome Abbreviations: AIDP - Acute Inflammatory Demyelinating Polyneuropathy, AMAN - Acute Motor Axonal Neuropathy, AMSAN - Acute Motor-Sensory Axonal Polyneuropathy.

**Figure 2:**
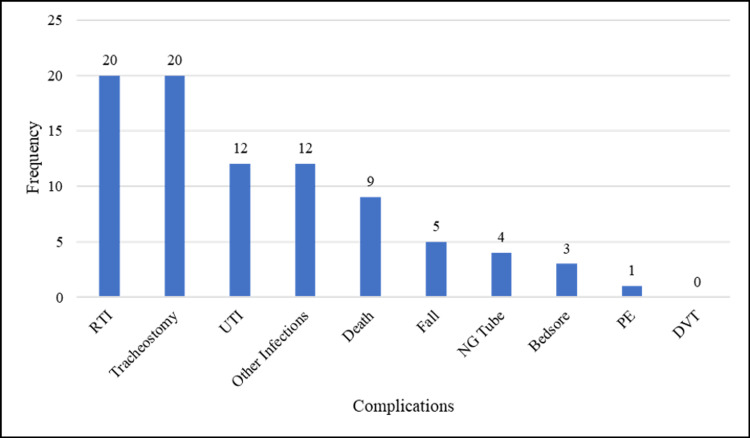
Complications in patients with Guillain-Barré syndrome Abbreviations: RTI - Respiratory Tract Infection; UTI - Urinary Tract Infection; NG Tube - Nasogastric Tube; PE - Pulmonary Embolism; DVT - Deep Vein Thrombosis.

CSF examination was performed in 70 (81.4%) cases. A summary of the CSF findings and other diagnostic evaluation, electrophysiological patterns, and treatment of GBS are shown in Table 2. NCS were performed in 79 (91%) patients, among whom AMAN variety of GBS was the commonest electrophysiological type affecting 41 (51.9%) of the patients, followed by acute inflammatory demyelinating polyneuropathy (AIDP) as shown in Figure 1. A large majority of patients 70 (81.3%) received IVIG. A total of 41 (47.7%) patients required ICU admission, whereas three (3.5%) patients died in the hospital. The management and outcome including discharge disposition and discharge disability are shown in Table 3. In-hospital complications are shown in Figure 2.

**Table 3:** Management and outcome of patients with Guillain-Barré syndrome (N=86)

Variables	Categories	N (%)
Acute (first) treatment	Intravenous immunoglobulin (IVIG)	70 (81.3)
	Plasma exchange (PE)	12 (14.0)
	No acute treatment	4 (4.7)
Second treatment option	IVIG after PE or PE after IVIG	7 (8.1)
Hospital length of stay	< 1 week	18 (21.4)
	1 – 2 weeks	21 (25.0)
	3 – 4 weeks	13 (15.5)
	>4 weeks	32 (38.1)
ICU admission	Admitted	41 (47.7)
ICU length of stay	< 1 week	26 (66.7)
	1 – 2 weeks	2 (5.1)
	3 – 4 weeks	5 (12.8)
	>4 weeks	6 (15.4)
Discharge disposition	Home	16 (20.5)
	Rehabilitation	33 (42.3)
	Transfer to other hospital	17 (21.8)
	Transfer to long term facility	2 (2.6)
	Still admitted at 3 months	5 (6.4)
	Discharge against medical advice	5 (6.4)
Modified Rankin Scale at discharge	0–2 (mild disability)	21 (24.4)
	3–5 (moderate to severe disability)	52 (60.5)
	6 (death)	3 (3.5)
	Data not available	10 (11.6)
Hughes Disability Score at discharge	0 (Normal)	1 (1.2)
	1–3 (Mild disability)	44 (51.2)
	4–5 (moderate to severe disability)	28 (32.6)
	6 (death)	3 (3.5)
	Data not available	10 (11.5)
Hughes Disability Score at 1 year	0 (Normal)	19 (22.1)
	1–3 (mild disability)	24 (27.9)
	4–5 (moderate to severe disability)	5 (5.8)
	6 (death)	4 (4.7)
	Data not available	34 (39.5)

**Figure 3:**
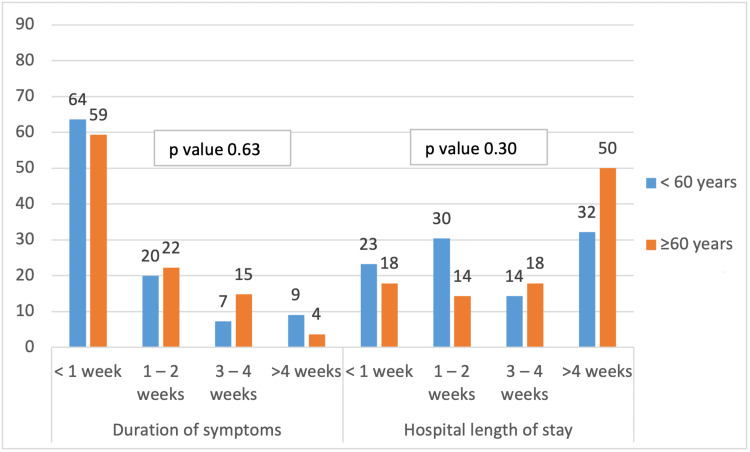
Duration of symptoms prior to presentation and hospital stay compared in patients according to age

Gender and age-related comparisons of management and outcomes are shown in Table 4 and Figure 3. There was no significant difference in the rate of ICU admission, management and discharge disposition between males and females. Females were more likely to have higher disability at the time of discharge (p=0.01). Patients less than 60 years of age were more likely to be admitted to the ICU (p-value < 0.01).

**Table 4:** Comparison of management and outcomes based on gender and age

Variables	Categories	Male (n= 55)	Female (n=31)	P-value
Duration of symptoms	< 1 week	36 (66.7%)	15 (53.6%)	0.21
	1–2 weeks	12 (22.2%)	5 (17.9%)	
	3–4 weeks	4 (7.4%)	4 (14.3%)	
	>4 weeks	2 (3.7%)	4 (14.3%)	
Hospital length of stay	< 1 week	11 (20.4%)	7 (23.3%)	0.58
	1–2 weeks	12 (22.2%)	9 (30.0%)	
	3–4 weeks	9 (16.7%)	4 (13.3%)	
	>4 weeks	21 (38.9%)	8 (26.7%)	
ICU admission	Admitted	25 (45.5%)	16 (51.6%)	0.58
IVIG as first treatment	Not given	7 (13.2%)	5 (17.2%)	0.55
	1-3 days	39 (73.6%)	18 (62.2%)	
	4-7 days	6 (11.3%)	4 (13.8%)	
	>7 days	1 (1.9%)	2 (6.9%)	
Plasma exchange as first treatment	Not given	49 (89.1%)	25 (80.7%)	0.33
	Given	6 (10.9%)	6 (19.4%)	
Discharge disposition	Discharge to home	8 (16.3%)	8 (27.6%)	0.41
	All other discharge dispositions	41 (83.7%)	21 (69.0%)	
mRS at discharge	0–2	13 (27.1%)	8 (28.6%)	0.92
	3–5	32 (66.7%)	20 (71.4%)	
	6	3 (6.3%)	0 (0.0)	
Hughes disability score at discharge	0	1 (2.1%)	0 (0.0)	0.01
	1–3	32 (66.7%)	12 (42.9)	
	4–5	12 (25.0%)	16 (57.1%)	
	6	3 (6.3%)	0 (0.0)	
Age < 60 years			Age 60 years or above	p-value
ICU admission	Yes	28 (49.1%)	13 (44.8%)	<0.01

## Discussion

We present the largest single-center study of adult patients with GBS from the KSA. A recent multicenter study from eight tertiary care centers in Saudi Arabia reported findings on 156 patients with GBS [16]. Our retrospective investigation accumulated a decade’s experience at a major tertiary care center in Riyadh, the capital of KSA, with a rapidly growing population of 8.6 million persons in 2019 [17]. A 10-year study from Japan estimated an annual incidence of GBS of 0.42 cases per 100,000 persons [18]. A similar observation was made in a regional study from the middle east, estimating a mean incidence rate of 0.69 in 100,000 persons in a local province [19]. Most recently, a nationwide study in China estimated the incidence of GBS in adults to be 0.829 per 100,000 persons [20]. In contrast, a larger systemic review including studies exclusively from North America and Europe, estimated an annual incidence of approximately one to two per 100,000 person-years [21]. The gender difference of GBS patients found in our study was consistent with national, regional, and international findings [13,14,16,19,21]. We found the classical male predominance in our study with a ratio of 1.7:1, which is consistent with the most recent national study at a ratio of 1.6:1, and the earlier studies [13-16]. An exception to this was one old study reporting a slight female predominance of 1:1.27, back in 1991 [12]. Regional studies have reported a similar male predominance in Oman and Iran, at a ratio of 1.75:1, and 1.5:1, respectively [19,22]. The mean age at onset was 49.5±17.5 years which is similar to an older local study that investigated GBS cases across all age groups, reporting a mean age of 45 at disease onset [14]. In contrast, a recent national study reported the age of disease onset with a median of 38 [16]. Our findings were also consistent with one regional study from Oman reporting a mean age at the onset of 42.69 years [22]. On the other hand, a systematic review reported a lower age of onset more frequently in individuals in their 20s and 30s among the Arab population [23]. Globally, the incidence of GBS is reported to be higher at 50 years of age and older, and its risk increases by 20% for every 10-year increase in age [20,21,24]. The majority of our patients were Saudi nationals. One regional study included only the citizens, whereas another study reported citizens to be the vast majority of patients with GBS [19,22]. This may be related to selection bias as Saudis have easier access to tertiary care compared to non-Saudi residents. Additionally, our center caters primarily to the National Guard soldiers and their families, hence, the majority of patients in our center were Saudi nationals. In our cohort, a large majority of patients were married whereas smoking was not very common. The prior local studies on adults with GBS have not reported on the marital status, or smoking prevalence of their patients [13,14,16].

An antecedent infection is a common finding in the natural history of GBS since approximately two-thirds of patients complain of symptoms four to six weeks prior to the disease onset [9]. More than half of our patients had reported some kind of infection including upper respiratory tract infection, urinary tract infection, or diarrhea prior to the disease manifestation. Our results were consistent with a recent national study reporting respiratory tract infections (39.1%) and diarrhea (27.8%) as common preceding infections [16].

Many of our patients had premorbid risks of vascular disease such as diabetes mellitus, hypertension, and coronary artery disease. Almost half of our patients had hypertension, it might be of significance to highlight the effects of the proceeding vascular comorbidities since one case has reported hypertensive encephalopathy triggering GBS [25]. At the time of GBS diagnosis, 30.2% of our patients had diabetes mellitus, 12.8% had coronary artery disease, and 8.1% had hypothyroidism. Conversely, a regional, multicenter study reported only one patient having diabetes mellitus, 20.1% having hypertension, and lower values of chronic comorbidities compared to our sample population [19]. This may be due to the overall older age of our patients. About 7% of our patients reported having a previous neuromuscular disease, whereas 8.1% reported a history of a previous neuropathy. Similar to our findings, a study from Iran reported the presence of neurological disease in 6.3% of their patients [19].

Sensorimotor weakness is the commonest complaint among GBS patients. Typically, patients present with an ascending, symmetrical pattern of weakness [9], and almost 87% of our patients showed a similar distribution. The majority of local and regional studies have shown a distribution similar to ours [13,14,23]. Only seven of our patients were found to have pure lower limb paraparesis, a finding that is identical to an older local study done in Riyadh [13]. Around 60% of our patients complained of numbness, which is higher than numerous GBS studies done on both adults and pediatric populations in Saudi Arabia, and neighboring countries [13-15,19,22]. Pain in GBS imposes a major burden on both patients and healthcare systems, be it limb or back pain. Backache was reported in 38.6% of our patients, considerably higher than a regional study in Iran where only 2.8% complained of back pain [19]. Many patients with GBS suffer from disease progression to cranial nerves causing facial weakness, dysarthria, and other bulbar signs [9]. Facial weakness is the commonest cranial nerve dysfunction among GBS patients in Arab countries [23]. Our patients also showed involvement of cranial nerves including facial weakness in 32.6% of patients. However, these values are lower than some Saudi studies, yet it is considerably higher than a recent regional study from Iran [12,16,19]. Unfortunately, no Saudi study has reported laryngeal involvement of GBS patients in adults. Luckily, one recent regional study reported the presence of dysarthria in 4% of their patients, whereas our patients had a five times higher prevalence of it at 20.9% [19]. Similarly, another regional study reported a low observation of cranial nerve involvement, where only 6.8% of their patients developed cranial nerve involvement [22]. Compared to a recent pediatric GBS study from our tertiary care center, our adult GBS patients had a higher prevalence of facial weakness [15]. Ocular abnormalities were frequent in our cohort as 17.4% had ophthalmoplegia/ophthalmoparesis, which is higher than a recent national study at 12.4% [16]. About 12.8% of our patients complained of diplopia, which is higher than a recent regional study reporting double vision in only 3.4% of GBS patients [19].

Autonomic dysfunction was seen in almost 20% of our patients, which is more frequent than in any other studies in Saudi Arabia or other Arab countries [13,16,23]. Bladder involvement was also seen in nearly 7% of our patients which is more frequent than reported in other recent regional studies [19,22]. The majority of our patients had areflexia or hyporeflexia, which is consistent with studies done in Arabic countries [12-14,16,23]. About 34.9% of our patients complained of dyspnea, which is considerably higher than the most recent national study [16]. Respiratory weakness was observed in 27.9% of our patients, which is slightly higher compared to most recent Saudi and regional studies [13,16,19,22]. Higher levels of fasting plasma glucose have been reported to be associated with bulbar signs and autonomic dysfunction, dyspnea, and the need for mechanical ventilation [26]. This might correspond with the increased frequency of aforementioned manifestations in our study, as almost one-third of our patients had diabetes mellitus at the time of developing GBS.

Diagnostic investigations, such as CSF analysis and electrophysiological studies, are imperative for the diagnosis of GBS [9]. AMAN variant of GBS was the commonest subtype observed in 51.9% of our patients. Multiple Saudi, and regional studies have shown a predominance of the AIDP variant of GBS [16,19,22,23]. There is no clear explanation for this finding. Almost 47.7% of our patients required ICU admission. ICU admissions were more common in patients with axonal variants of GBS, as reported by a recent Saudi study, where 42.3% of patients presenting with axonal variants, required ICU admission [16]. The higher need for ICU admission in our patients may be related to the axonal variant of GBS. Many of the studies that reported AIDP as their commonest subtype, had lower ICU admissions compared to ours [19,22]. Older age and ICU admission are poor prognostic indicators of GBS [27]. Interestingly, younger patients in our study were significantly more likely to be admitted to the ICU. Low mortality has been reported in prior Saudi studies. In our cohort as well, only three of the patients died in the hospital [16]. In our cohort, females were more likely to have a higher degree of disability at the time of discharge. There was no clear explanation for this finding as the females did not differ from males in other demographic or clinical features.

One of the strengths of our study is that this is the largest series from a single center in Saudi Arabia. The data included many variables that had not been previously well reported. Additionally, we were able to make comparisons based on gender and age stratification.

There are several limitations to our study. Being a single-center study, it could not determine the incidence of GBS in Saudi Arabia. The retrospective nature of the study made it difficult to obtain several outcome variables. The discharge information did not systematically include the disability outcome scales and some of the data had to be inferred from the available information. Long-term follow-up was not available for a large majority of the patients.

## Conclusions

Our patients with GBS were found to be slightly older than previously reported in the region, and the most common type of GBS observed was the AMAN variant. Additionally, our study revealed that our GBS patients exhibited similar clinical features and CSF findings as reported in national, regional, and international studies. Patients under the age of 60 were more likely to require admission to the ICU, whereas females were more likely to have a more severe disability. Our study was the first to focus solely on Saudi patients, providing a detailed description of concurrent morbidities.
